# The Ethics of Proximity: Bedside Ethics and the Contribution of NICU Nurses to Ethical Deliberation

**DOI:** 10.3390/healthcare14203474

**Published:** 2026-10-16

**Authors:** Clara Todini, Simone S. Masilla, Costanza Raimondi, Irene Cardinali, Andrea Ponzio, Barbara Corsano, Dario Sacchini, Pietro Refolo, Antonio G. Spagnolo

**Affiliations:** 1 Department of Healthcare Surveillance and Bioethics, Section of Bioethics and Medical Humanities, Università Cattolica del Sacro Cuore, 00168 Rome, Italy; salvatoresimone.masilla@unicatt.it (S.S.M.); costanza.raimondi1@unicatt.it (C.R.); andrea.ponzio02@icatt.it (A.P.); dario.sacchini@unicatt.it (D.S.); pietro.refolo@unicatt.it (P.R.); antoniogioacchino.spagnolo@unicatt.it (A.G.S.); 2 Fondazione Policlinico Universitario A. Gemelli IRCCS, 00168 Rome, Italy; irene.cardinali@policlinicogemelli.it; 3 Clinical Ethics Consultation Service, Fondazione Policlinico Universitario A. Gemelli IRCCS, 00168 Rome, Italy; barbara.corsano@unicatt.it; 4 Research Center for Clinical Bioethics & Medical Humanities, Università Cattolica del Sacro Cuore, 00168 Rome, Italy

**Keywords:** bedside ethics, nursing, clinical ethics, NICU, ethical decision-making, clinical ethics consultation

## Abstract

**Background/Objectives**: Ethical decision-making in neonatal intensive care units (NICUs) requires the integration of clinical evidence, prognostic evaluation, and relational perspectives to determine the newborn’s best interests. Pellegrino’s concept of bedside ethics emphasizes the moral significance of proximity and the knowledge generated through the clinical encounter. However, the specific contribution of NICU nurses to ethical deliberation remains insufficiently explored. This conceptual article aims to explore the contribution of NICU nurses to multidisciplinary ethical deliberation and shared decision-making, focusing on how bedside presence and relational proximity inform ethical reflection and clinical decisions. **Methods**: A literature-informed approach was conducted to synthesize empirical studies, theoretical contributions, and academic literature addressing the ethical role of NICU nurses. A literature search was conducted between April and July 2026 in PubMed and Scopus using terms related to neonatal intensive care, nursing, ethics, ethical decision-making, bedside ethics, shared decision-making, clinical ethics consultation, and moral distress. Eligible publications included empirical studies, reviews, theoretical papers, and one professional position statement. Sources were analyzed through an iterative conceptual synthesis. **Results**: Four interconnected dimensions of NICU nurses’ ethical contribution were identified: the epistemic, dialogical and advocacy, integrative, and educational roles. Nurses’ continuous bedside presence generates longitudinal knowledge of the newborn’s clinical condition, suffering, comfort, and family dynamics, enriching ethical deliberation beyond biomedical assessments. **Conclusions**: NICU nurses should be recognized as essential participants in ethical deliberation. Their proximity to the newborn represents not only an ethical responsibility but also an epistemic resource. Systematic inclusion of bedside nurses in multidisciplinary ethics discussions and Clinical Ethics Consultation processes is essential to promote ethically robust, relational, and patient-centered decision-making.

## 1. Introduction

Every clinical act is, by its very nature, an ethical act: every therapeutic decision, diagnostic intervention, and care-related choice necessarily carries moral implications that directly affect the well-being of the individual receiving care. Through his concept of *bedside ethics*, Edmund D. Pellegrino advanced a vision of clinical ethics firmly grounded in the lived experience of caring and in the concrete encounter between the healthcare professional and the patient. According to Pellegrino, moral questions do not arise from the mere abstract formulation or the mechanical application of ethical principles; rather, they emerge from the clinical encounter itself and from the moral responsibility inherent in this interpersonal relationship [1]. The patient’s bedside therefore assumes a meaning that extends beyond its physical and spatial dimension, emerging as the privileged locus of ethical deliberation, where the vulnerability of the person receiving care confronts healthcare professionals with a moral responsibility that demands the prudent integration of clinical expertise, relational competence, and ethical judgment. From this perspective, bedside ethics can be understood as a renewed expression of the ethics of care, grounded in relationality, sustained by presence and proximity, and shaped by the moral commitment that arises within the clinical encounter [2].

Within this ethical framework, nursing assumes a distinctive role because of its continuous presence at the patient’s bedside. This proximity places nurses in a privileged position within the therapeutic relationship and suggests a potentially unique contribution to ethical deliberation, particularly in contexts characterized by high clinical and moral complexity. Accordingly, nursing can be conceived as a concrete expression of Pellegrino’s conception of the moral purpose of clinical medicine—*the pursuit of the patient’s good*—by translating ethical commitment into a continuous and relational presence through which the patient’s interests, dignity, and well-being are protected throughout everyday clinical practice [1].

If, from Pellegrino’s perspective, the clinical relationship constitutes the foundation of ethical practice, neonatal care raises distinctive ethical questions that, at least superficially, appear to challenge this paradigm. Unlike the competent adult patient, the newborn is unable to articulate needs, preferences or values, and lacks the decisional autonomy that traditionally underpins the clinician–patient relationship. In this context, vulnerability (already a central concept in Pellegrino’s ethical framework) reaches its fullest expression, rendering the newborn entirely dependent upon the moral and professional responsibility of those entrusted with his or her care. Yet, paradoxically, it is precisely this apparent relational asymmetry that underscores the distinctive relevance of bedside ethics in neonatal practice. The absence of verbal communication does not imply the absence of a therapeutic relationship; rather, it requires a different way of recognizing and responding to the patient’s needs.

Among healthcare professionals, nurses are uniquely positioned to fulfill this relational and interpretative role because of their continuous presence at the newborn’s bedside [3].

In neonatology, the nurse–patient relationship inevitably extends to include the parents, who are not only the child’s surrogate decision-makers but also their primary advocates and essential partners in the care team. Consistent with this perspective, the recent literature on family-centered care emphasizes that parental presence at the newborn’s bedside, active participation in care, and shared decision-making are associated with improved clinical outcomes, a stronger parent–infant bond, and a higher ethical quality of care [4,5]. Within this relational model, the nurse’s continuous presence therefore assumes a dual significance: on the one hand, it enables the interpretation of the newborn’s needs through careful and continuous clinical observation; on the other, it facilitates the development of a therapeutic alliance with parents, fostering their involvement in care provision and decision-making processes.

The relevance of *bedside ethics* becomes particularly evident in the Neonatal Intensive Care Unit (NICU), where healthcare professionals are frequently required to make decisions characterized by a high degree of clinical and ethical complexity. Although advances in neonatal intensive care have significantly improved the survival of extremely preterm infants and newborns with severe conditions, they have also increased the frequency of difficult decisions concerning the initiation, continuation, limitation, or withdrawal of life-sustaining treatments [6].

Within this context, ethical deliberation extends beyond the technical feasibility of intervention to include an assessment of the proportionality of care and the newborn’s best interests, a complex and contested concept that may encompass multiple and potentially competing considerations, including anticipated benefit, suffering, treatment burden, quality of life, parental values and preferences, family circumstances, and clinical uncertainty. As contemporary literature emphasizes, therapeutic proportionality cannot be determined solely by prognostic or technological considerations but requires a careful evaluation of the expected benefits and burdens of treatment, including the infant’s experience of pain, suffering, and quality of life [7]. These considerations naturally lead to the broader theme of neonatal clinical ethics, where moral reflection is translated into concrete decision-making in some of the most ethically challenging situations encountered in clinical practice.

Such complexity has progressively fostered the development of multidisciplinary models of ethical deliberation, in which interdisciplinary dialogue represents an essential tool for integrating diverse expertise, perspectives, and values, with the aim of pursuing the best interests of the child and their family [8]. Recent literature also highlights a growing reliance on Clinical Ethics Consultation (CEC) services (where available) within NICUs as a means of supporting the management of cases characterized by particular moral and decision-making complexity. Multidisciplinary meetings have become one of the main tools of CECs in the neonatal intensive care setting, reflecting the growing recognition that ethically complex decisions require early interdisciplinary collaboration, particularly for life-limiting and life-threatening neonatal conditions [9].

Within these deliberative processes, neonatologists, nurses, other medical specialists, psychologists, clinical ethics consultants, and, whenever possible, the patient’s parents, share clinical, prognostic, and care-related information with the aim of reaching transparent and ethically justifiable decisions. Ethical deliberation therefore assumes a collaborative nature, in which the value of a decision does not depend solely on the scientific accuracy of the available information, but also on the team’s ability to integrate diverse professional perspectives and to consider the patient as a whole. In this respect, *shared responsibility* represents one of the defining principles of family-centered neonatal care [10], requiring continuous negotiation of responsibilities between healthcare professionals and parents, as well as the genuine integration of each participant’s contribution to the deliberative process. Accordingly, the quality of decision-making is enhanced when the deliberative process recognizes and incorporates the contributions of all healthcare professionals directly involved in patient care [11].

Despite the increasing adoption of multidisciplinary ethical deliberation within NICUs, little attention has been paid to the distinctive contribution that nurses make to these deliberative processes through their continuous bedside presence and their unique knowledge of the newborn. The aim of this study is to explore and critically analyze the distinctive contribution of NICU nurses to multidisciplinary ethical deliberation and shared decision-making in neonatal intensive care. Specifically, the study examines how nurses, through their continuous bedside presence and their privileged relationship with both the newborn and the family, contribute to the recognition, interpretation, and management of ethical dilemmas, as well as to the deliberative processes through which ethically justifiable clinical decisions are reached.

## 2. Materials and Methods

This conceptual article was developed through a literature-informed approach aimed at exploring the ethical contribution of nurses to bedside ethical deliberation in the neonatal intensive care setting. Relevant literature was identified through searches conducted in PubMed and Scopus using combinations of keywords related to neonatal intensive care, nursing, ethics, ethical decision-making, bedside ethics, ethical deliberation, shared decision-making, moral distress, and clinical ethics support. PubMed and Scopus were selected as the primary databases because of their complementary coverage of biomedical, clinical, nursing, healthcare, and interdisciplinary literature. PubMed was particularly relevant for identifying literature concerning neonatal intensive care, nursing, and clinical ethics, whereas Scopus provided broader multidisciplinary coverage across healthcare, nursing, social sciences, and bioethics. In developing the conceptual framework, particular attention was paid to the contextual relevance of the literature. Evidence derived from NICU-specific studies was preferentially used to support claims concerning neonatal nursing practice and the ethical particularities of neonatal intensive care. Literature from broader nursing or other clinical contexts was considered when it addressed concepts of wider theoretical relevance, such as advocacy, relational knowledge, ethical sensitivity, or participation in ethical decision-making. The search was conducted between April and July 2026 and was used to identify literature relevant to the development and contextualization of the conceptual argument. the literature search served as a non-systematic, purposive process to identify relevant empirical, theoretical, and professional literature capable of informing the central conceptual perspective of the article. Literature was considered relevant when it addressed the neonatal intensive care context and provided meaningful insights into the nursing role in relation to ethical decision-making, multidisciplinary deliberation, shared decision-making, or the ethical contribution of nurses. No restriction was applied to study design, in order to allow the inclusion of diverse forms of literature relevant to the conceptual development of the topic. Given the heterogeneous nature of the literature considered, sources were not treated as equivalent in terms of evidentiary weight. No formal critical appraisal tool or hierarchy of evidence was applied, as the purpose of the literature search was not to conduct a systematic synthesis of evidence. Rather, sources were considered according to their relevance to the conceptual focus of the article, their methodological nature, and their contribution to the development of the argument. Empirical studies were primarily used to provide evidence regarding clinical and professional experiences, whereas reviews were used to contextualize broader areas of knowledge. Theoretical papers and professional position statements were used mainly to inform the ethical, conceptual, and professional dimensions of the discussion. These different forms of literature were therefore used in complementary, rather than interchangeable, ways to support and contextualize the conceptual framework.

Rather than conducting a formal qualitative thematic analysis, the literature was subjected to an iterative conceptual synthesis. Relevant publications were read and examined for recurring concepts concerning the role and contribution of NICU nurses to ethical decision-making, multidisciplinary deliberation, communication, advocacy, coordination, and education. Relevant concepts and observations were progressively identified, compared across sources, and clustered according to their conceptual similarities.

Through this iterative process, four overarching dimensions of the nursing role were developed: **1. epistemic, 2. dialogical and advocacy, 3. integrative, and 4. educational**. These dimensions emerged through the interaction between the concepts identified in the literature and the authors’ interpretation of their relevance to bedside ethics.

The authors’ experiential and professional perspective was used to interpret and integrate the literature, rather than being treated as empirical evidence. The four dimensions therefore represent a literature-informed conceptual framework proposed by them, rather than findings generated through a formal qualitative coding procedure. All authors except a single one are actively involved as clinical ethics consultants within the CEC Service of the Fondazione Policlinico Universitario A. Gemelli IRCCS (Rome, Italy), where they regularly participate in multidisciplinary ethics meetings concerning complex clinical cases. In addition, the first author is a NICU nurse at Bambino Gesù Children’s Hospital in Rome, Italy, and is currently pursuing a PhD in Bioethics and Medical Humanities at Università Cattolica del Sacro Cuore, bringing both clinical and academic expertise to the study. These complementary experiences provided contextual insight into the interpretation of the literature and the conceptual development of the review while not constituting an independent source of evidence. This positioning provides relevant experiential insight into the ethical challenges encountered in neonatal practice, but may also shape the questions considered salient and the interpretation of the literature. The resulting framework therefore emerged from the interplay between published literature and the authors’ professional experience and reflections, with the literature serving to support, contextualize, and critically develop the central argument concerning nurses’ proximity to patients and families and their potential contribution to ethical deliberation at the bedside.

## 3. Results

The literature-informed conceptual synthesis led to the identification of four interconnected dimensions through which the contribution of NICU nurses to bedside ethics can be understood: epistemic, dialogical and advocacy, integrative, and educational. These dimensions should not be interpreted as discrete or independently occurring roles. Rather, they represent overlapping aspects of nursing practice that may converge in ethically complex clinical situations.

### 3.1. The Epistemic Role

Relevant literature has emphasized the distinctive contribution of NICU nurses to multidisciplinary ethical decision-making as deriving from the unique form of knowledge acquired through their continuous presence at the newborn’s bedside. In neonatal practice, bedside proximity assumes not only a caregiving function but also an epistemic one, as it enables nurses to acquire forms of knowledge that cannot be obtained through episodic clinical assessment alone. Unlike other healthcare professionals, whose evaluations are primarily based on intermittent clinical encounters, NICU nurses develop a longitudinal understanding of the infant through continuous observation and daily caregiving [12]. This sustained presence enables them to recognize subtle changes in physiological stability, behavioral responses, comfort, pain, adaptation, parent–infant interactions, and emerging care needs that may not be readily captured through diagnostic investigations or technological monitoring alone.

This distinctive epistemic perspective allows nurses to contribute ethically relevant information that complements medical, diagnostic, and prognostic assessments. By integrating these observations into multidisciplinary deliberation, they help ensure that ethical decision-making is informed not only by biomedical evidence but also by the newborn’s lived clinical experience and the relational dimensions of care. Their contribution therefore extends beyond the delivery of nursing care, providing the multidisciplinary team with a more comprehensive understanding of the infant’s best interests and supporting clinical decisions that are both scientifically sound and ethically justified. This epistemic contribution becomes particularly relevant when multidisciplinary teams evaluate the proportionality of life-sustaining treatments, as the assessment of proportionality depends not only on prognosis and technological effectiveness but also on a comprehensive understanding of the newborn’s lived clinical condition.

Defining the ethical limits of intervention requires distinguishing between what can be done and what ought to be done, particularly in light of the continuous technological advances characterizing contemporary neonatal intensive care. While innovations in neonatal medicine have significantly improved the survival of critically ill newborns, they have also increased the complexity of ethical decision-making, as the mere availability of life-sustaining technologies does not necessarily justify their initiation or continuation.

Clinical decisions must therefore be guided by an ethical evaluation of whether a treatment remains proportionate to the expected benefits and consistent with the newborn’s best interests, rather than by technological capability alone [13]. Closely related to proportionality is the concept of treatment burdensomeness, which refers to the overall burden that medical interventions place upon the patient. In neonatal intensive care, burdensomeness cannot be directly reported by the newborn but must instead be inferred through careful clinical observation and validated pain and stress assessment tools. Indicators such as persistent physiological instability, repeated painful procedures, behavioral signs of stress, impaired neurobehavioral regulation, and prolonged dependence on invasive life-support technologies provide important evidence regarding how treatment is experienced by the infant. Because NICU nurses are continuously present at the bedside, they are uniquely positioned to recognize and longitudinally monitor these indicators. Their observations therefore constitute ethically relevant knowledge that complements medical and prognostic assessments, enabling the multidisciplinary team to evaluate not only whether a treatment remains physiologically effective but also whether the *burdens* it imposes remain proportionate to its expected benefits and to the newborn’s best interests [14].

The evaluation of burdensomeness also contributes to distinguishing therapeutic proportionality from medical futility, two concepts that are frequently associated but should not be considered synonymous. A treatment may be medically futile when it is no longer capable of achieving its intended physiological goal or when there is no reasonable expectation that it will alter the patient’s clinical trajectory. Conversely, a treatment may retain physiological effectiveness while nevertheless becoming ethically disproportionate because the burdens it imposes significantly outweigh its foreseeable benefits. Accordingly, the epistemic contribution of NICU nurses may provide a valuable perspective to deliberations concerning the withholding or withdrawal of life-sustaining treatments, ensuring that decisions are informed not only by judgments of physiological effectiveness but also by careful consideration of the infant’s suffering, overall clinical condition, and best interests [6].

### 3.2. The Dialogical and Advocacy Role

Previous contributions offer insights into the role of NICU nurses as key facilitators of ethical dialogue within multidisciplinary decision-making. Owing to their continuous interaction with the newborn, the family, and the healthcare team, nurses occupy a unique position that enables them to promote communication, mutual understanding, and the integration of different perspectives throughout the deliberative process. One of their primary contributions is to translate bedside observations into information that is clinically meaningful and ethically relevant. By interpreting the newborn’s non-verbal expressions of pain, comfort, distress, and adaptation, nurses ensure that the infant’s lived experience is represented during multidisciplinary discussions rather than being overshadowed by diagnostic or prognostic considerations alone and thereby enrich the ethical quality of deliberation [15]. Nurses also facilitate dialogue between healthcare professionals and parents by reducing informational asymmetry. Through continuous communication and the translation of complex medical language into understandable terms, they enable parents to participate meaningfully in discussions regarding goals of care and treatment decisions, thereby fostering a process of authentic shared decision-making [16].

This *dialogical role* becomes particularly important in multicultural settings, where nurses frequently adapt communication according to families’ cultural, religious, and social values. By promoting culturally sensitive communication, they facilitate mutual understanding between families and healthcare professionals, helping ensure that ethical deliberation remains attentive not only to clinical evidence but also to the values, beliefs, and relational context of the newborn and family [17,18]. The multiplicity of relationships they maintain and the numerous contexts in which nurses operate render them knowledgeable and competent across several domains, enabling them to mediate effectively among the actors involved and to reconcile the different perspectives that emerge during multidisciplinary deliberation. By facilitating dialogue among all parties involved, NICU nurses contribute to a deliberative process in which clinical evidence, parental perspectives, and ethical considerations can be integrated into decisions that are both scientifically sound and ethically justified while remaining centered on the newborn’s best interests.

By fostering this continuous exchange of information and perspectives, NICU nurses also fulfill a fundamental *advocacy role*. Because newborns are unable to express their preferences or participate directly in decision-making, nurses play a crucial role in ensuring that their interests, dignity, experiences of suffering, and evolving care needs are consistently represented throughout the deliberative process. Through the continuous interpretation of bedside observations and their close therapeutic relationship with both the infant and the family, nurses help prevent ethical deliberation from being driven exclusively by diagnostic, prognostic, or technological considerations, ensuring that the voice of the most vulnerable patient remains ethically present in multidisciplinary discussions. This advocacy role extends beyond the protection of patients’ rights, encompassing the professional responsibility to safeguard the newborn’s best interests by integrating clinical evidence with the relational and human dimensions of care, describing nurses as important advocates for patients and families in ethically complex clinical situations [19].

### 3.3. The Integrative Role

The existing scholarship suggests that the meaningful inclusion of NICU nurses in multidisciplinary ethical deliberation contributes not only to improving the quality of ethical decision-making but also to strengthening the moral integrity and collaborative functioning of healthcare teams. Conversely, the exclusion or marginalization of nurses from formal ethical discussions represents one of the major contributors to moral distress within neonatal intensive care settings [20]. A recurring finding across the reviewed studies is that nurses, despite being continuously involved in patient care and possessing unique bedside knowledge, are frequently assigned a marginal role during discussions with physicians and families. This discrepancy between professional responsibility and limited influence over ethical decisions generates feelings of powerlessness, frustration, and moral distress [21].

The literature considers that structured multidisciplinary meetings and CEC services provide opportunities for nurses to actively contribute to ethical deliberation, allowing their epistemic and dialogical contributions to be fully integrated into decision-making. When nurses participate as equal members of the multidisciplinary team, individual moral burden is progressively transformed into shared moral responsibility, reducing professional suffering while simultaneously enhancing the ethical quality, transparency, and legitimacy of clinical decisions [22].

These findings suggest that the inclusion of NICU nurses in ethical deliberation should not be regarded merely as a strategy for reducing moral distress, but as an essential condition for fostering genuine interdisciplinary collaboration and ensuring ethically robust decision-making.

### 3.4. The Educational Role

The literature has described NICU nurses as playing a distinctive educational role throughout the process of ethical deliberation. Unlike episodic clinical encounters, nursing care is characterized by continuous interaction with both the newborn and the family. This sustained presence enables nurses to accompany parents throughout the clinical journey, progressively fostering their understanding of the newborn’s condition, the goals of treatment, and the ethical implications of complex clinical decisions.

Education therefore becomes an integral component of nursing care rather than a separate activity. Through repeated conversations, clarification of clinical information, and continuous emotional support, nurses help families progressively develop the knowledge and confidence necessary to participate meaningfully in ethical deliberation and shared decision-making. Their educational role is not limited to explaining medical information but extends to facilitating ethical understanding, enabling parents to appreciate the values, uncertainties, benefits, and burdens underlying different therapeutic options.

The educational role of NICU nurses also extends to the multidisciplinary team. Through everyday clinical practice, bedside discussions, and reflective exchange with colleagues, nurses continuously contribute to the development of ethical awareness, encouraging critical reflection on ethically challenging situations and promoting a shared culture of ethical responsibility.

Specific preparation may be relevant to the effective fulfilment of this facilitative role. Early and continuous education in clinical ethics, communication, and ethical deliberation equips nurses with the competencies necessary to recognize ethical dilemmas, facilitate discussion, and support ethically justified decision-making [23,24]. Conversely, inadequate ethical preparation has been associated with greater professional distress, reduced confidence in ethical deliberation, poorer support for families, and lower quality of care [13].

## 4. Discussion

### 4.1. The Epistemic Value of Nursing Proximity

The central proposition of this article is that proximity may have an epistemic dimension within bedside ethics. In the NICU, nurses spend sustained and repeated periods of time with infants and families, often across different phases of illness and treatment. Through this continuity of care, they may acquire forms of experiential knowledge that are not necessarily captured by clinical measurements or by episodic encounters with the patient.

This knowledge may include subtle changes in the infant’s behaviour and responses, patterns of comfort or distress, the perceived burden of treatment, and changes in family interactions, expectations, and concerns. Such knowledge is inherently relational and situated: it develops through repeated encounters and through the nurse’s ongoing participation in the care relationship.

From an ethical perspective, this suggests that nursing proximity should not be understood solely as a characteristic of bedside care, but also as a potential source of knowledge relevant to ethical deliberation. The nurse may therefore contribute to ethical discussion not only by representing the patient’s or family’s perspective, but by bringing clinically situated and relational knowledge into the deliberative process.

This does not imply that nurses possess an inherently superior ethical perspective, nor that proximity alone guarantees ethically preferable judgement. Rather, the argument is that proximity may generate a distinctive form of knowledge that can complement biomedical, prognostic, and parental perspectives within multidisciplinary deliberation. Recognizing this epistemic dimension may therefore broaden the understanding of what constitutes relevant knowledge in neonatal ethical decision-making.

### 4.2. From Nursing Proximity to Ethically Relevant Knowledge

The ethical relevance of nursing proximity lies not simply in the amount of time nurses spend at the bedside, but in the type of knowledge that may emerge from sustained engagement with the infant and family. Continuous observation across time can provide access to changes, patterns, and relational dynamics that may remain less visible during episodic clinical encounters. Nurses may observe subtle variations in the infant’s responses to treatment, manifestations of comfort or distress, the burden associated with ongoing interventions, and changes in parental understanding, expectations, and emotional responses. These observations are not, in themselves, ethical judgements; however, they may become ethically relevant when clinical decisions involve questions of proportionality, treatment goals, quality of life, treatment burden, or the newborn’s best interests.

This distinction is particularly important for bedside ethics. In Pellegrino’s account, ethical reflection is rooted in the concrete clinical encounter rather than occurring independently from the realities of patient care. The ethical significance of clinical knowledge therefore depends not only on abstract principles or biomedical facts, but also on an understanding of the particular patient, the clinical relationship, and the circumstances in which decisions are made. Within this framework, nurses’ situated knowledge may constitute one component of the knowledge required for ethically informed deliberation.

The argument can therefore be understood as a progression from proximity to knowledge, from knowledge to ethical relevance, and from ethical relevance to deliberative inclusion. If nurses’ sustained presence allows them to develop knowledge that is relevant to understanding the infant’s condition, treatment experience, family relationships, and evolving goals of care, excluding this perspective from deliberation may leave part of the ethically relevant clinical picture unrepresented. This provides a conceptual, rather than empirically demonstrated, justification for creating institutional conditions in which nurses can contribute to multidisciplinary ethical deliberation.

Such a proposition does not imply that nurses possess a privileged or superior ethical perspective, nor that their presence alone determines the ethically appropriate course of action. Rather, it suggests that ethical deliberation may be strengthened when different forms of clinically relevant knowledge are brought together. Nursing proximity can therefore be understood as an epistemic resource whose ethical value lies in its potential to complement biomedical, prognostic, parental, and other professional perspectives.

The present article suggests that the contribution of NICU nurses to multidisciplinary ethical deliberation extends far beyond the implementation of clinical care. Rather than representing an additional professional perspective within multidisciplinary meetings, nurses contribute a distinct form of ethical knowledge generated through their continuous bedside presence [25]. This epistemic contribution constitutes the foundation upon which their dialogical, advocacy, integrative, and educational roles develop, making nursing participation an essential component of ethically grounded decision-making rather than an optional complement to medical expertise.

Our findings provide a contemporary interpretation of Pellegrino’s concept of bedside ethics. While Pellegrino located the moral origin of clinical ethics in the encounter between the clinician and the patient, the present review suggests that, within neonatal intensive care, this encounter is most continuously experienced by nurses. Consequently, bedside ethics should not be understood solely as the ethical responsibility of individual clinicians, but also as an epistemic resource that multidisciplinary deliberation requires in order to remain genuinely centered on the patient’s good [1,2].

These findings also have practical implications for NICU organization. Multidisciplinary ethics meetings and CEC services should systematically include bedside nurses as active participants in deliberation. Furthermore, undergraduate and continuing education programs should strengthen competencies in clinical ethics, communication, and shared decision-making, enabling nurses to fulfil their ethical responsibilities effectively.

The conceptual framework emerging from this review also carries important implications for the organization of ethical decision-making within NICUs. If the distinctive contribution of nurses derives from the epistemic value of their continuous bedside care, their participation in multidisciplinary ethical deliberation should not depend on individual initiative or local organizational culture, but should instead be recognized as an integral component of ethically sound clinical governance. Multidisciplinary ethics meetings, end-of-life case conferences, and CEC services should therefore systematically include bedside nurses as active participants throughout the deliberative process. Their involvement should extend beyond the communication of clinical observations or the implementation of decisions already made, encompassing full participation in the identification of ethical problems, the exploration of alternative courses of action, the assessment of therapeutic proportionality, and the formulation of ethically justified recommendations.

Recognizing nurses as equal members of ethical deliberation also requires a cultural shift in the understanding of professional roles within neonatal intensive care. Traditionally, ethical decision-making has often been perceived as primarily belonging to physicians, while nursing participation has frequently remained consultative or supportive [26]. The present review suggests that such an approach underestimates the unique epistemic contribution generated through continuous bedside care and risks depriving multidisciplinary deliberation of information that is essential for evaluating the newborn’s best interests. Ethical decision-making should therefore be understood as a genuinely collaborative process in which different forms of professional knowledge are not hierarchically ordered but integrated according to their complementary contributions [9]. Within this framework, nursing participation represents neither an expansion of professional authority nor a redistribution of medical responsibility, but rather the recognition that ethically well-founded decisions require the integration of multiple perspectives that no single professional can provide independently.

These considerations also have important implications for CEC services. Ethics consultation within NICUs should move beyond a predominantly physician-centered model toward a genuinely interdisciplinary deliberative process in which nurses participate from the earliest stages of case analysis. Their continuous knowledge of the infant’s clinical evolution, behavioral responses, comfort, suffering, and family dynamics provides ethically relevant information that complements medical diagnosis and prognosis, allowing ethics consultations to develop a more comprehensive understanding of the case [27]. Incorporating nursing perspectives systematically into ethical consultation may therefore contribute not only to improving the quality and legitimacy of recommendations but also to strengthening trust, transparency, and shared responsibility among all professionals involved in neonatal care.

The findings likewise highlight the need to strengthen ethics education throughout nursing training and professional development. The competencies required to participate effectively in multidisciplinary ethical deliberation cannot be assumed to arise solely from clinical experience but require structured educational preparation. Undergraduate nursing curricula should therefore provide systematic education in clinical ethics, ethical reasoning, communication, shared decision-making, and family-centered care, while postgraduate and continuing professional development programmes should offer opportunities to refine these competencies through case-based learning, simulation, ethics rounds, and participation in multidisciplinary deliberative activities. Such educational initiatives would enable nurses not only to recognize ethical dilemmas more confidently, but also to contribute actively to ethical analysis, facilitate dialogue with families, and support multidisciplinary teams confronted with morally complex decisions [28].

Strengthening ethical competence may also have important organizational benefits. Several studies included in this article suggest that greater preparation in clinical ethics is associated with increased confidence in participating in ethical discussions, more effective communication with families, and reduced experiences of moral distress among healthcare professionals [29]. Conversely, insufficient ethical training may limit nurses’ willingness to express ethically relevant concerns, reduce their perceived legitimacy within multidisciplinary discussions, and ultimately compromise both professional well-being and the quality of ethical deliberation. Investing in ethics education should therefore be regarded not only as an educational priority, but also as an organizational strategy capable of promoting moral resilience, interdisciplinary collaboration, and ethically sustainable clinical practice within NICUs.

Ultimately, translating these findings into organizational practice requires acknowledging that the ethical quality of decision-making depends not only on the availability of advanced technologies or specialized medical expertise, but also on the effective integration of the professional most continuously present at the patient’s bedside. Institutional policies that formally recognize nurses’ participation in multidisciplinary ethics meetings, provide protected time for ethical deliberation, and support ongoing education in clinical ethics may therefore represent important steps toward strengthening the ethical governance of neonatal intensive care. In this perspective, the inclusion of bedside nurses should not be regarded as an optional organizational refinement but as a necessary condition for ensuring that ethical deliberation remains genuinely patient-centered, relational, and responsive to the best interests of the newborn. It is important to distinguish the evidence identified in the literature from the normative implications proposed in this conceptual analysis. The literature provides evidence that nurses in NICU are closely involved in the care of infants and families, participate in communication and decision-making processes, and encounter ethically challenging situations in everyday practice. Building on these observations, we argue that nurses’ sustained proximity to infants and families may constitute an important source of knowledge that could contribute to ethical deliberation. This interpretation should not be understood as an empirically established claim or as evidence that nurses necessarily make different or superior ethical judgements. Rather, it represents a conceptual proposition arising from the intersection of the available literature and the authors’ clinical and scholarly experience. From this perspective, we propose that greater recognition of nurses’ contribution to ethical deliberation may be considered in clinical ethics practice and multidisciplinary decision-making.

### 4.3. Implications for Bedside Ethical Deliberation

If nursing proximity is understood as a source of situated knowledge, its relevance extends beyond routine clinical care. In ethically complex situations, nurses may bring to multidisciplinary deliberation observations and relational insights developed through sustained contact with the infant and family. This may be particularly relevant when ethical decisions concern proportionality of treatment, quality of life, treatment burden, goals of care, or end-of-life decision-making.

The conceptual implication is not that nurses should replace other professional or parental perspectives, but that their experiential knowledge may be more explicitly recognized as one component of the ethical evidence considered during deliberation. This perspective also supports a broader understanding of bedside ethics, in which ethical knowledge is not produced exclusively through formal consultation or abstract principles, but may also emerge from situated clinical relationships and everyday care practices.

The figure below (Figure 1) provides a visual conceptualization of the key concepts discussed above, illustrating how nurses’ continuous bedside proximity translates into experiential, longitudinal, and relational knowledge, which in turn shapes their ethical contribution and role within multidisciplinary deliberation and ethically informed neonatal decision-making.

## 5. Limitations of the Study

This review has several limitations that should be considered when interpreting its findings.

First, as a conceptual paper, it was designed to provide a conceptual synthesis rather than a quantitative assessment of the available evidence. Consequently, no formal appraisal of methodological quality or meta-analysis was performed.

Second, the literature addressing the ethical contribution of NICU nurses remains heterogeneous in terms of study design, conceptual frameworks, and clinical contexts, limiting direct comparison across studies.

Third, because much of the available evidence is qualitative or theoretical, some of the proposed conceptual relationships require empirical validation. Future research should investigate how nursing participation in multidisciplinary ethical deliberation influences decision-making processes, family involvement, moral distress, and patient-centered outcomes within neonatal intensive care settings.

The fourth limitation concerns the scope of the literature search. Although PubMed and Scopus provided broad coverage of biomedical, nursing, healthcare, and interdisciplinary literature, discipline-specific databases such as CINAHL were not searched. Consequently, some nursing-specific literature may not have been identified. This limitation should be considered when interpreting the literature-informed conceptual analysis. However, the purpose of the search was not to provide an exhaustive or systematic synthesis of the evidence, but to identify relevant literature supporting the development and contextualization of the conceptual framework.

## 6. Conclusions

This article suggests that the contribution of NICU nurses to multidisciplinary ethical deliberation extends far beyond the implementation of clinical care. Their continuous bedside presence generates a distinctive form of knowledge that enriches ethical deliberation by integrating clinical evidence with the newborn’s lived experience, relational context, and best interests. The epistemic, dialogical, advocacy, integrative, and educational dimensions identified in this review should therefore be understood not as independent professional functions but as complementary expressions of the ethical value of proximity in neonatal care.

By revisiting Pellegrino’s concept of *bedside ethics*, this review proposes that, within neonatal intensive care, the nurse’s continuous presence at the bedside represents not only an ethical responsibility but also an indispensable epistemic resource for multidisciplinary decision-making. Ethical deliberation is strengthened when nursing knowledge is systematically integrated with medical, prognostic, and family perspectives, allowing decisions to reflect both scientific evidence and the human realities of caring for critically ill newborns.

The existing literature indicates that nurses’ continuous proximity to infants and families provides them with a distinctive perspective on clinical situations, relationships, communication, and experiences of suffering and treatment burden. Building on this literature, we propose that such experiential and relational knowledge may represent a valuable, yet not always fully recognized, contribution to multidisciplinary ethical deliberation.

These considerations should be understood as a conceptual proposition. Further empirical research is needed to explore how NICU nurses contribute to ethical deliberation in practice, how their perspectives are incorporated into multidisciplinary decision-making, and under which conditions their participation may meaningfully support ethically complex neonatal care.

These findings support the need to promote organizational models that recognize bedside nurses as full participants in multidisciplinary ethical deliberation and CEC services.

Observational studies of multidisciplinary meetings, evaluations of CEC processes, and the development of tools to assess the impact of nursing contributions on decision-making, family involvement, moral distress, and patient-centered outcomes may provide important evidence for translating the ethics of proximity into increasingly effective models of neonatal ethical governance. Recognizing this contribution may broaden the understanding of what counts as relevant knowledge in neonatal ethics.

## Figures and Tables

**Figure 1:**
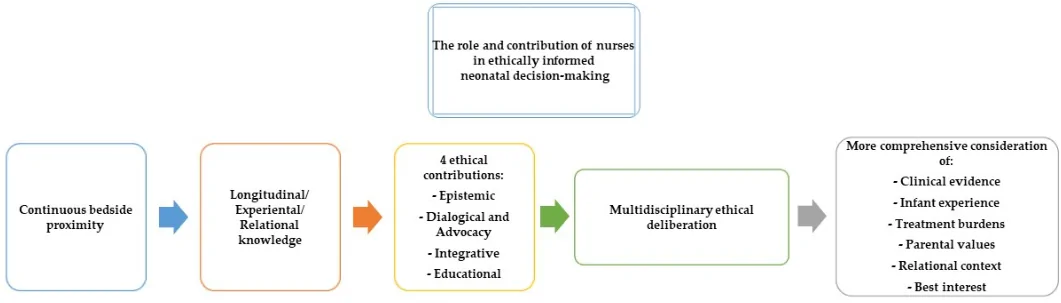
Visual conceptualization of the role and contribution of nurses in ethically informed neonatal decision-making.

## Data Availability

The data presented in this study are available in the article.

## Abbreviations

The following abbreviations are used in this manuscript:

NICU	Neonatal Intensive Care Unit
CEC	Clinical Ethics Consultation
